# Workgroup Report: National Toxicology Program Workshop on Hormonally Induced Reproductive Tumors—Relevance of Rodent Bioassays

**DOI:** 10.1289/ehp.10135

**Published:** 2007-07-05

**Authors:** Kristina A. Thayer, Paul M. Foster

**Affiliations:** National Institute of Environmental Health Sciences, National Institutes of Health, Department of Health and Human Services, Research Triangle Park, North Carolina, USA

**Keywords:** animal models, breast, endocrine, hormone, mammary gland, National Toxicology Program, ovary, prostate, reproductive tumors, species differences, testis

## Abstract

The National Toxicology Program (NTP) is currently reviewing its research portfolio as part of its efforts to implement the NTP Roadmap to achieve the NTP Vision for the 21st century. This review includes a recent workshop, “Hormonally Induced Reproductive Tumors—Relevance of Rodent Bioassays,” held 22–24 May 2006, that was organized to determine the adequacy and relevance to human disease outcome of rodent models currently used in the 2-year bioassay for four types of hormonally induced reproductive tumors (ovary, mammary gland, prostate, and testis). In brief, none of the workshop’s breakout groups felt the currently used models are sufficient. For some types of tumors such as prostate, no adequate animal models exist, and for others such as ovary, the predominant tumors in humans are of different cellular origins than those induced by chemicals in rodents. This inadequacy of current models also applies to the testis, although our more complete understanding of the responses of Leydig cells to hormonal changes in rats may prove predictive for effects in humans other than cancer. All breakout groups recommended that the NTP consider modifying its testing protocols (i.e., age at exposure, additional end points, etc.) and/or using alternative models (i.e., genetically engineered models, *in vitro* systems, etc.) to improve sensitivity. In this article we briefly review the workshop’s outcome and outline some next steps forward in pursuing the workshop’s recommendations. Breakout group reports and additional information on the workshop, including participants, presentations, public comments and background materials, are posted on the NTP website.

The National Toxicology Program (NTP) hosted the workshop “Hormonally Induced Reproductive Tumors—Relevance of Rodent Bioassays” on 22–24 May 2006 in Raleigh, North Carolina, to discuss the adequacy of rodent models used in the 2-year bioassay by the NTP for detecting certain reproductive tumors. This workshop is the third in a series of activities associated with the NTP Roadmap to critically evaluate the NTP testing program and determine whether any refinements or new strategies are needed to maximize its impact on public health (NTP 2005c). More than 100 people from academia, industry, government, and nonprofit organizations attended the workshop, including an invited panel of 55 scientists with expertise in endocrinology, cancer biology, reproductive toxicology, statistics, and other related fields. The workshop opened with a series of presentations on each of the target tumors from the perspective of clinical and epidemiologic studies, mode(s) of action, and rodent models. To address the workshop’s objectives, the invited panels also met in tumor site-specific breakout groups and summarized their discussions in a plenary session. The workshop agenda, presentations, background materials, roster of the invited panel and other attendees, and public comments can be found on the NTP website (http://ntp.niehs.nih.gov; see “Meetings & Workshops” or directly at http://ntp.niehs.nih.gov/go/18592).

The workshop’s overall objective was to determine the utility and relevance to human disease outcome of experimental rodent models for evaluating four types of reproductive tumors (ovary, mammary gland, prostate, and testis) with known or presumed hormonal etiologies. The NTP is interested in these issues because of concern that current rodent models, including those used by the NTP, may not adequately detect carcinogens that act via the endocrine system or other nongenotoxic modes of action. Tumors of the mammary gland, ovary, prostate, and testis were selected for evaluation because of their significant human morbidity and mortality and concern that currently used models are not optimal for addressing these tumor types. In some cases, the modes of action thought to cause these types of tumors in rodents are not believed relevant to humans. For example, atrazine-induced mammary gland tumors in the Sprague-Dawley rat are attributed to precocious reproductive senescence that results from atrazine’s effects on neuroendocrine function [U.S. Environmental Protection Agency (EPA) 2000]. In certain strains of rats, reproductive senescence in females is characterized by a state of persistent estrus and high levels of estrogen, which may increase the risk of tumor formation in estrogen-responsive tissues. This mode of action is generally not considered relevant to humans because circulating levels of endogenous estrogen decrease during menopause.

In other cases, the rodent species and strains currently used in NTP chronic bioas-says (F344/N rat and B6C3F_1_/N mouse) are suspected to be poor models because they either do not develop a specific type of tumor or have a high spontaneous tumor incidence. Either situation can make detecting a chemically induced effect difficult. For example, in NTP chronic bioassays, prostate tumors are rarely observed in control animals and have never been clearly associated with a chemical exposure, suggesting that the current models are not sensitive for detecting potential human prostate carcinogens. However, this problem is not specific to the F344/N rat and B6C3F_1_/N mouse, as no conventional rodent models are considered ideal to assess chemically induced prostate cancer (NTP 2006e). At the other extreme are testicular Leydig cell tumors in the F344/N rat. The spontaneous incidence in control animals is so high (∼ 70–90%) that it can be difficult to detect a statistically significant increase above background (NTP 2005a). The conventional rodent models may also be poor predictors of human response because of significant differences between rodents and humans in tumor prevalence, anatomy, or tumor histology.

Following are brief summaries of the break-out group discussions, which focus on the overall utility of currently used rodent models and what is known regarding endocrine-related modes of action. Presentations and breakout group reports that provide the foundation for these summaries are available on the NTP website (see previously mentioned websites). It is worth noting that all the breakout groups struggled with some of the same general issues. For example, they were unsure how to evaluate the predictiveness of rodent models for identifying potential human carcinogens given the limited number of agents adequately evaluated in humans. In addition, the scientific literature for both humans and rodents often does not allow distinguishing between endocrine hormone-induced and endocrine system-mediated roles in the etiology and progression of tumorigenesis.

## Breakout Group Discussions

### Testis

#### Background information: predominant tumor types and role of the endocrine system

The NTP selected testicular tumors for evaluation because incidence of the disease is increasing in men in certain regions of the world and the there is concern that current NTP rodent models (F344/N rat and B6C3F_1_/N mouse) are not optimal for identifying potential human testicular carcinogens (NTP 2005a). More than 90% of testicular tumors in men are germ cell tumors, and only approximately 2% are interstitial or Leydig cell tumors. In contrast, germ cell tumors are rarely (at most) observed in rodents, whereas spontaneous Leydig cell tumors are extremely common in the F344/N rat. The high background incidence of Leydig cell tumors in the F344/N rat (∼ 70–90%) makes detecting a statistically significant increase in these tumors in treated animals extremely difficult. Only two (ethylbenzene and isoprene) of the more than 500 chemicals tested in NTP chronic bioassays show clear evidence of causing testicular adenomas in the F344/N rat. Other rat strains, such as the Sprague-Dawley and Wistar Han, have a lower spontaneous incidence of Leydig cell tumors (∼ 5%), making them potentially more sensitive models (NTP 2005a). The B6C3F_1_/N mouse has a very low incidence of spontaneous Leydig cell tumors (< 1%) but also appears to be resistant to developing chemically induced testicular tumors, as none have been identified in NTP studies. Leydig cell tumors in other strains of mice can be induced chemically (particularly by estrogenic agents), but generally mice are resistant to the wide variety of agents known to induce these tumors in rats, particularly the Sprague-Dawley and Wistar (Cook et al. 1999).

In both rats and mice, luteinizing hormone (LH) acts as a promoter in the development of Leydig cell clusters, hyperplasia, and tumors. Differences in response to LH between the rat and human may partially explain the greater sensitivity of rats to develop Leydig cell tumors compared with that in humans. In rats, chemically induced Leydig cell tumors are usually observed after treatment with compounds that increase LH. In humans, a syndrome exists that can be used for comparative purposes. Familial male precocious puberty (FMPP) stems from a mutation in the LH receptor, resulting in constitutive activation. Men with this disorder undergo puberty at around 4 years of age, although they have normal fertility and lifespan. Individuals with FMPP exhibit Leydig cell hyperplasia but are not known to have a higher incidence of Leydig cell tumors. Human Leydig cells have lower numbers of LH, gonadotropin-releasing hormone agonist (GnRH), and prolactin receptors per cell than rodent Leydig cells, which may translate into a reduced sensitivity of response to ligands for these receptors. The mouse differs from both the rat and human. The same compounds that induce Leydig cell tumors in rats do not typically have this effect in mice. In mice, Leydig cell tumors have been induced by estrogen agonists and antagonists that may be acting directly by inducing Leydig cell proliferation and indirectly by increasing LH levels and LH receptor concentrations.

Low levels of androgens or disorders that result in decreased androgen response are associated with testicular tumors in rats and men. The antiandrogen flutamide is a potent inducer of Leydig cell tumors in rats, presumably through causing a rise in LH secretion. The incidence of Leydig cell tumors in men who have a defective androgen receptor that makes them insensitive to androgens is considerably higher than for men without this syndrome (2.3% vs. ∼ 0.00004%). Human germ cell tumors are considered to have an *in utero* origin, and androgens are required for progression of the transformed gonocyte (fetal germ cell) or carcinoma *in situ* (CIS) to a germ cell tumor in humans. The incidence of germ cell tumors is also higher in men with androgen insensitivity syndrome, providing support for the role of androgens in development of germ cell tumors. The seemingly complex relationship between androgens and germ cell tumors may be a function of the relatively high amount of androgen required for normal reproductive tract development and differentiation of germ cells versus the lower amount needed for germ cell proliferation. Lowered androgen may aid the production of CIS cells because of “faulty” differentiation but still be present in sufficient amount to allow proliferation of the CIS cells once formed. The extent to which gonadal cell differentiation is normal in rodents with complete androgen insensitivity syndrome has yet to be adequately addressed.

#### Conclusions and recommendations

Despite differences in tumor incidence and hormone response, rodent models are still considered useful. The characteristics of Leydig cell tumors and their diagnostic criteria are generally similar for rodents and men, although there may be differences in terminology. For example, the term “Leydig cell micronodule” in humans is often equated to small adenomas in rodents (Holm et al. 2003). Germ cell tumors are too rare in rodents to evaluate the similarities between rodents and men. The breakout group recommended that the NTP explore the 129 mouse strain, which is predisposed to developing male germ cell tumors, as a possible model for germ cell tumors in men.

CIS have both stem- and germ-cell properties and originate from undifferentiated gonocytes because of poor function of Sertoli and/or Leydig cells during testis development. CIS is considered part of the testicular dysgenesis syndrome where environmental and genetic factors can alter Sertoli cell function, leading to reduced semen quality and CIS, or decrease Leydig cell function, leading to hypospadias and cryptorchidism. For this reason, some members of this breakout group suggested that Leydig cell nodules/hyperplasia in the rat might serve as a surrogate marker for germ cell tumors in men. However, the break-out group believed that evaluation of Leydig cell nodules/hyperplasia for this purpose would be most informative in a cancer study that included *in utero* exposure. Currently, the NTP does not undertake *in utero* exposure routinely as part of its cancer studies; therefore, the critical windows for development of potential testicular dysgenesis in rodents are not evaluated. The breakout group also emphasized that testicular cancer is a reproductive disease and men with this disease are more likely to have cryptorchidism and low sperm counts. For this reason, the group suggested that identification of similar outcomes in rodents might serve as early predictors of testicular germ cell tumor induction.

### Mammary gland

#### Background information: predominant tumor types and role of the endocrine system

The NTP included evaluation of mammary gland tumors in the workshop because breast cancer is the most common type of cancer in women, affecting approximately 1 in 7. Another reason for selecting this target site is concern that the NTP’s current rodent models (the F344/N rat and B6C3F_1_/N mouse) are not ideal for identifying potential human breast carcinogens.

Both similarities and differences exist between rodents and humans in terms of tumor characteristics and diagnostic criteria. Fibroadenoma is the predominant lesion in some strains of rat but is not considered a pre-malignant lesion in humans. Adenoma and carcinoma in rats are considered to be relevant to breast cancer in humans, but human metastases can be hard to model, as mammary gland carcinomas in rodents rarely metastasize. Premalignant lesions (e.g., atypical hyperplasia) can be observed in both rats and humans. In contrast, premalignant lesions in the mouse do not parallel pathological changes in humans.

Historically, incidences of mammary gland tumors in control animals are lower in female mice compared with female rats (NTP 2005a). In fact, the high background incidence of mammary gland tumors, particularly fibroadenomas (∼ 40–45%), in female F344/N rats complicates interpretation of the NTP bioassay. From one perspective it could be argued that this high background incidence mimics the relatively common occurrence of breast cancer in women (lifetime risk is 12.7%). However, from a statistical perspective, high background rates of tumors in control animals generally decrease the ability to detect an exposure-related effect. In addition, when a statistically significant tumor effect is found in test animals relative to concurrent controls, the effect may not be considered exposure related if it falls within the range observed in historical controls. From a biological perspective, even though fibroadenomas are clinically relevant, they are a benign tumor and may not be predictive of carcinoma in women.

It is clear from both epidemiologic and experimental animal studies that breast tumors can have an endocrine component. In women, an increased exposure to endogenous or synthetic estrogens increases the risk of developing the disease. Factors that lead to greater lifetime exposure to endogenous estrogens (i.e., early menses, late menopause, nulliparity, post-menopausal obesity) are associated with elevated risk. Older age at first full-term birth (≥ 30 years vs. < 20 years) also increases the risk of developing breast cancer. Exposures to synthetic estrogens or pharmaceuticals that contain combinations of estrogens and progesterones such as diethylstilbestrol, oral contraceptives, and hormone replacement therapy are also linked to increased risk. The role of other “reproductive” hormones such as prolactin, progesterone (when not combined with estrogen), and androgens thought important in stimulating mammary cancer in certain rodent models is less clear in breast cancer for women.

The breakout group emphasized that nonsteroidal and nonpituitary hormones such as insulin-like growth factor 1 (IGF-1) and insulin may play a role in the etiology and progression of breast cancer. What is less clear is whether endocrine-active compounds act as tumor initiators or promoters. For example, ovariectomized rats do not develop mammary gland tumors even when treated with potent chemical carcinogens as positive control compounds (Russo and Russo 1998). This finding suggests that ovarian hormones are acting as promoters. The breakout group noted that tumors may arise from endocrine alterations and genotoxicity as well as epigenetic effects.

The endocrine differences between rodents and women can complicate the interpretation of a positive bioassay finding. For example, prolactin is the predominant driver of tumor development in F344 rats; however, whether this hormone is a causative agent in humans is not clear. The breakout group noted that premature ovarian failure caused by exposure to a particular agent could produce a false negative tumorigenic effect for the mammary gland since a loss of ovarian hormones and function results in mammary tissue regression.

#### Conclusions and recommendations

In the absence of an ideal model, the existing rodent models are useful for identifying the potential ability of a compound to induce biological change and serve a useful screening function to identify potential carcinogens. More specifically, the rat is considered to be a better model than the mouse for the purpose of identifying potential human carcinogens because mice are generally more resistant to developing chemically induced mammary gland tumors. For example, the Carcinogenic Potency Database (CPDB) identifies only 24 chemicals associated with mammary gland tumors in mice compared with 102 in rats (Gold et al. 2001). In the traditional NTP bioassay where both rats and mice are tested for each compound, 11 chemicals are identified as having “some” or “clear” evidence of mammary gland tumors in mice, whereas 27 are identified as such in rats (and 4 are positive in both). However, without a better understanding of the underlying biology and the relevance of changes in rodents to changes in humans, the full predictive value of these models cannot be realized.

Furthermore, the standard NTP bioassay may miss important opportunities for identifying compounds capable of causing breast cancer. The group recommended considering experimental designs that address relevant exposure windows (e.g., *in utero* exposures, exposures during puberty, exposures before a first full-term pregnancy) and that distinguish between pre- and postmenopausal risk to detect more agents of potential human concern.

### Prostate

#### Background information: predominant tumor types and role of the endocrine system

Prostate tumors were chosen for evaluation at the workshop because prostate cancer is the most common cancer in males in the United States and the third most common cancer worldwide. In addition, it is clear that current, conventional rodent models are not useful for identifying prostate carcinogens. Unlike the human condition, spontaneous prostate cancer is very rare in rodents, including the F344/N rat and B6C3F_1_/N mouse used by the NTP (2005a). In a survey of almost 4,550 rats and mice used as controls in NTP inhalation or feed studies only 1 carcinoma and 17 adenomas were detected (NTP 2006e). No substances have been identified as causing prostate tumors in NTP studies. There is likewise currently little compelling evidence for environmental agents causing prostate cancer in humans.

In addition to differences in spontaneous tumor incidences, the anatomy of the prostate gland in humans and rodents is considerably different (i.e., the rodent prostate is lobular and the human prostate is not). Also, the diagnostic criteria and terminology used to describe prostate histopathology in experimental biology, toxicology, and clinical medicine differ between rodents and humans. For example, NTP and other veterinary toxicology pathologists do not use the diagnostic term “prostatic intraepithelial neoplasia” or “Gleason scores” that are used in experimental biology and in clinical practice because proliferative prostate lesions are so rare in their rodent models.

Prostate tumors can be induced in rodents via treatment with several compounds, including androgens, estrogens, and certain mutagenic agents. With respect to endocrine modulation, perinatal treatment with estrogen or long-term treatment with testosterone or testosterone and estradiol can induce prostate adenocarcinomas and carcinomas although the tumor type and incidence are species, strain, and protocol dependent. The evidence in humans is less clear regarding whether an altered endocrine environment can cause prostate tumors although numerous studies link elevated levels of IGF-1 with prostate cancer. Androgens appear to play a “permissive” role in the development of prostate tumors.

#### Conclusions and recommendations

Despite the differences in prostate anatomy and pathology between humans and rodents, the breakout group felt it would be prudent to consider prostatic inflammation and hyperplasia in rodent models relevant for human diseases of the prostate such as benign prostatic hyperplasia (BPH) and prostate tumors. The group also suggested NTP investigate the various genetically engineered models that have been developed to study factors related to the pathogenesis of experimental prostate cancer. These models, however, are generally driven by molecular mechanisms not necessarily relevant or specific to human prostate cancer (e.g., promotion of SV40 viral inserts). To improve the ability to detect environmental factors that may contribute to prostate cancer, the breakout group encouraged the NTP to consider dosing animals in the early postnatal period during prostate duct development and to search for rodent strains more sensitive to development of prostate cancer. More extensive histopathology of the prostate was also recommended, as chemically induced preneoplastic changes may have value for assessing human carcinogenic potential.

### Ovary

#### Background information: predominant tumor types and role of the endocrine system

Although the life-time risk of developing ovarian cancer is not high (∼ 1.5%), it is the most lethal female reproductive system cancer. The absence of a screening test and a lack of knowledge about the symptoms of ovarian cancer result in a diagnosis that typically does not occur until the later stages of the disease when survival drops to 20–30%.

Unfortunately, differences in human and rodent ovarian pathophysiology limit the applicability of conventional rodent models for understanding the causes, progression, and therapeutic interventions of this disease. Approximately 90% of ovarian cancers in women originate from the surface epithelium. In contrast, granulosa cell tumors are the most common type of rodent ovarian tumor, and spontaneous epithelial cell tumors are only rarely noted. With exception of the mutagen 7,12-dimethylbenz[*a*]anthracene (DMBA), chemical exposures are not known to cause epithelial ovarian tumors in rodents. In addition, spontaneous and nonmutagenic chemically induced rodent ovarian tumors are generally benign. The low incidence of spontaneous ovarian tumors in rats and mice may limit the utility of the rodent bioassay for detecting possible human ovarian carcinogens (NTP 2005a). Historically the incidences for various types of ovarian tumors in control animals for both the F344/N rat and B6C3F_1_/N mouse are typically < 1% (NTP 2005a, 2006e). Only 10 chemicals show “some” or “clear” association with ovarian tumors in NTP studies, and none are reported carcinogenic in the rat (NTP 2006e), suggesting that ovarian tumors are not readily induced by chemical exposures in NTP model systems.

The breakout group believed the evidence suggests that epithelial ovarian tumors in women can result from an altered endocrine environment. For example, the risk of developing ovarian cancer increases around the time of menopause and for women taking estrogen-only hormone replacement therapy. In contrast, increases in the number of live births and in oral contraceptive use are associated with decreased risk. Elevated levels of circulating gonadotropins are clearly associated with stromal tumor development based on studies using genetically engineered rodent models such as the inhibin-α knockout, estrogen receptor-α knockout, and transgenic animals that over-express LH-β. Ovarian germ cell tumors are not associated with an altered endocrine environment; however, the mechanisms of toxicity for compounds that induce ovarian tumors in rodents are typically unknown or unclear.

#### Conclusions and recommendations

Despite significant differences in ovarian tumors between humans and rodents, the breakout group thought certain ovarian observations in rodent bioassays should be assumed relevant for humans. For example, elevated circulating gonadotropins and loss of ovarian hormones should be considered relevant because they are similar to the endocrine status of menopausal women when most ovarian cancers occur. Other findings such as ovarian atrophy are good predictors of ovarian failure but not ovarian cancer. The breakout group also identified several preneoplastic events that NTP could consider evaluating for predictiveness of human response, including loss of contact inhibition, stratification of the surface epithelium, loss of p53, loss of phosphatase and tensin homolog (PTEN), and overexpression of phospho-AKT in epithelial cells. The group discussed several recently developed genetically engineered models [i.e., p53 and retinoblastoma (Rb) conditional knock-out, K-ras activation, and PTEN loss] that may be useful but have not been evaluated for their predictiveness for human risk.

## Major Workshop Recommendations and NTP Perspectives

### Use alternative models (i.e., genetically engineered, in vitro, etc.) to develop sensitive models for detecting specific types of tumors

#### NTP perspective

The appeal of using alternative disease-specific models is obvious. In many cases, these models are assumed to be better at identifying carcinogens (at least in rodents) than the conventional models used by the NTP. However, the NTP must maintain a balance between retaining the original goal of the chronic bioassay as a general screen for identifying potential carcinogens and investing resources into developing and using disease-specific models. Clearly, it is not feasible to study every compound in both conventional and disease-specific models; however, there is a compromise strategy. One of the major goals of the NTP Roadmap is to identify markers that are indicative of disease progression and incorporate them into prechronic or other preliminary studies. Findings from these studies might then trigger using a specialized model or study design. This approach is not new for the NTP but rather would expand its current practice. For example, the NTP is using the transgenic mouse model rasH2 in 6-month cancer studies for a number of polybrominated diphenyl ether (PBDE) congeners because the thyroid is a known target for the PBDEs and these transgenic models detect chemically induced thyroid tumors (NTP 2006f).

Identifying appropriate alternative models is especially important for prostate and ovarian cancers because the conventional rodent models are not predictive for these tumor types. The NTP has used the transgenic adenocarcinoma of the mouse prostate (TRAMP) model to address the impact of dietary antioxidants and dietary restriction on prostate cancer development (Suttie et al. 2005; Tam et al. 2006). Current studies generally focus on the ability of substances to inhibit prostate cancer development, and the NTP needs perhaps to place a greater emphasis on exploring the use of the TRAMP and other models to identify substances that enhance prostate carcinogenesis.

### Include additional endocrine responsive end points

#### NTP perspective

All the breakout groups had suggestions for preneoplastic responses or molecular markers that could potentially be included in prechronic studies. With respect to hormone-mediated tumors, the NTP is most interested in markers that are predictive for tumorigenesis and relevant to human disease. Suggested endocrine responsive assays and end points include *in vitro* assays to assess estrogen or androgen receptor binding, some of the short-term screens being evaluated by the U.S. EPA as part of its Endocrine Disruptor Screening Program (U.S. EPA 2006), and whole mounts of mammary glands. In addition, the mammary and prostate breakout groups both identified IGF-1 as an important stimulant and potentially useful marker of tumorigenic response. In the long-term, the NTP would like to include *in vitro* assays that screen for potential endocrine activity in its High Throughput Screening Initiative (see http://ntp.niehs.nih.gov/go/28213 for more information on this initiative). In the short-term, the NTP foresees more routine assessment of *in vivo* endocrine end points, such as whole mounts of mammary glands, when preliminary data suggest an endocrine mode of action or effect.

The NTP will consider incorporating additional end points into prechronic studies to better characterize end points of other environmentally induced diseases or biological processes related to disease etiology historically not addressed in NTP studies. For example, the goal of another NTP workshop, “Biomarkers for Toxicology Studies,” is to identify biomarkers for carbohydrate/lipid metabolism and lung and cardiac functions and evaluate their utility for inclusion in rodent toxicology studies (NTP 2006d). The NTP is still in the process of formally evaluating the most promising end points to include routinely in prechronic or other preliminary studies. The NTP intends to make these decisions over the next 2 years when additional NTP Roadmap activities are completed. As in the past, the inclusion of nonroutine end points will be done on a case-by-case basis.

It is worth noting that the NTP has previously used results from short-term studies to formulate predictions on carcinogenic potential. For example, the chemical diazoamino-benzene (DAAB) is considered a reasonably anticipated human carcinogen even though it has never been tested in a chronic bioassay, because metabolism studies showed that DAAB metabolizes to benzene and aniline, both known human carcinogens (Bordelon et al. 2005; NTP 2005b). Similarly, the NTP does not consider 1,4-butanediol to be carcinogenic in animals because it rapidly metabolizes to γ-hydroxybutyric acid, which did not show evidence of carcinogenic potential when tested by the NTP (Irwin 2006).

### Discontinue use of the F344/N rat

#### NTP perspective

The inadequacy of the F344/N rat as a model for testicular cancer was a major issue for the testicular tumor breakout group. The high background incidence of Leydig cell tumors in the F344/N makes it a very insensitive model for detecting chemically induced testicular tumors. Moreover, U.S. EPA testing guidelines do not recommend use of rats with low fecundity, such as the F344, for studies evaluating reproduction (U.S. EPA 1998). The NTP held a workshop in June 2006 specifically to address the issue of rodent strain and stock selections (NTP 2005a). The participants in that workshop strongly recommended that the NTP discontinues use of the F344/N because of its high background incidences of certain types of tumors (i.e., Leydig cell tumors and mononuclear cell leukemia), unresolved issues regarding its declining fertility, occurrence of sporadic seizures, and chylothorax. All these conditions are prevalent in the specific breeding colony maintained for NTP studies. Shortly after the workshop, the NTP discontinued use of the NTP F344/N rat in all new studies and began using a commercial source of the F344 (F344/NTac) as a short-term solution. The NTP originally intended to use an isogenic (inbred or F_1_ hybrid) rat strain to maximize reproducibility in tumorigenic response over time and facilitate genetic monitoring and interpretation of subsequent mechanistic studies (King-Herbert and Thayer 2006). However, the NTP is reconsidering this position so that it might use the same strain in studies for cancer, reproduction, or other types of toxicity. Using the same strain for all NTP studies would minimize the need to conduct multiple preliminary and toxicokinetic studies and enhance comparability across study end points.

The NTP recognizes that any particular strain or stock will likely have some undesirable characteristic (e.g., exceptionally low or high incidence for a specific tumor type, known insensitivity to certain exposures, lack of a historical data set, poor model for non-cancer studies). The issue of strain selection is only a question of which rodent model to use when very little or no toxicity data are available for a substance. As with end point selection, NTP studies are sufficiently flexible to allow the use of the most appropriate strain or model, and historically the NTP has followed this practice. For example, NTP studies might use a rat strain other than the F344/N if existing information indicates a need or considerable work has been done in another strain as long as there is no indication that the alternative model is inappropriate (NTP 2006b, 2006c). Previous work with an alternative strain can be used to ensure that the strain selected is responsive to a particular substance and can often negate the need for the NTP to conduct dose-finding studies. The NTP also tries to avoid using a strain known to be a poor model for a particular mode of action or target site for toxicity when such information is available for a given substance. For example, the NTP used the Sprague-Dawley rat in recent multigenerational studies for genistein because it is a better model for substances that may impact reproductive function than the F344/N rat (NTP 2006a). The major hesitation with moving away from routine use of the F344 rat is whether a new strain might be less sensitive. For this reason the NTP will obtain as much existing information as possible on comparative carcinogenic responses in the new strain(s) and the F344 rat.

The current top candidates to replace the F344/N rat are the Wistar Han and Sprague-Dawley rats, both outbred strains. The Wistar Han rat is appealing because it has excellent survival at 2 years of age, a large amount of data on control animals is available from its use in the pharmaceutical industry, and it has a relatively low spontaneous tumor profile. However, the NTP is concerned that the Wistar Han may not be a sensitive model for detecting mammary gland carcinogens (Ahlers et al. 1998) and toxicants that act through the aryl hydrocarbon receptor (Simanainen et al. 2004).

Although the NTP uses the Sprague-Dawley rat for reproductive studies, it previously was not seriously considered as a model for the chronic bioassay because available historical control data showed a low 2-year incidence for survival (∼ 40%). However, recent studies show that the stock of Sprague-Dawley maintained since the 1970s by the National Center for Toxicological Research (NCTR) of the Food and Drug Administration has an acceptable 2-year survival (62–86% for males and 43–64% for females; NTP 2006a). A second possibility is the Harlan Sprague-Dawley that has been used successfully in a number of recent NTP studies on 2,3,7,8-tetrachloro-dibenzo-*p*-dioxin (TCDD) and other “dioxin-like” chemicals (NTP 2006b, 2006c).

### Evaluate the importance of developmental programming in hormonally dependent issues leading to preneoplastic events and tumors

Overall, the breakout groups felt that *in utero* and lactational exposures could be added to the chronic bioassay depending upon what is known about the mode(s) of action or other endocrine-related effects of the particular substance under study. For detecting some tumor types such as testicular germ cell tumors, this recommendation was especially strong. The mammary gland group felt that any known developmental effects associated with a substance under study that involved endocrine tissues, steroid receptor binding, a change in mammary gland morphology, or altered timing of vaginal opening (an index of puberty) should trigger consideration of the use of *in utero* and lactational exposures. A number of participants suggested that the NTP consider using F_1_ animals from reproduction studies to better evaluate carcinogenic potential of perinatal exposures (as has been done recently for genistein) (Barrett 2006).

#### NTP perspective

NTP has long recognized the scientific appeal of including an *in utero* or perinatal exposure component in its chronic bioassays (Chhabra et al. 1993a, 1993b). Currently, these types of studies are generally undertaken when a substance is known to operate via a particular mode of action (i.e., endocrine) and/or there is evidence that fetuses and young animals might be especially susceptible. Of course, this type of information is unavailable for many substances studied by the NTP, leaving open the possibility that important toxicity findings are being missed by not addressing perinatal exposures. The NTP recently decided to incorporate perinatal exposure in its chronic carcinogenicity studies on a more routine basis. This means that future substances slated for carcinogenicity studies will include early in-life exposure unless there is a compelling reason to do otherwise (e.g., insufficient exposure to pregnant women and children, lack of placental transfer or accumulation in the fetus). In exceptional cases, the NTP may need to conduct multiple bioassays for a compound to address the carcinogenic potency for various periods of exposure (i.e., *in utero* only, *in utero* plus adult, and adult only). However, the cost of conducting these types of comparative studies is prohibitive for routine practice. NTP scientists are also considering other approaches to address early in-life exposures, including maintaining the F_1_ animals generated in reproductive studies for chronic studies and/or strategies to evaluate perinatal exposures in subchronic studies.

Any chronic or subchronic studies that include an *in utero* exposure period present challenging study design issues. For example, dose selection will probably be much more complicated than for the traditional chronic study. One objective in designing studies with perinatal exposure is to select doses that are sufficiently high to challenge the animals but not too high to terminate pregnancy, increase mortality, induce clinical signs of toxicity, or cause unacceptable body weight gain decrements. The doses administered to accomplish this objective may differ during critical periods in development and adulthood. Thus, the NTP may need to conduct multiple dose range-finding studies and make decisions regarding whether doses would change during the course of the study.

## Next Steps

As part of its implementation of the NTP Roadmap, the NTP sponsored four workshops during 2005 and 2006 to critically evaluate its testing program and determine whether any refinements or new strategies are needed to maximize its impact on public health (NTP 2005c). The NTP is committed to updating its research and testing programs to improve its ability to identify environmental substances that may pose a threat to human reproduction, development, and cancer through hormonally mediated mechanisms. The NTP has adopted some of the major recommendations from the hormonally induced tumors workshop and will begin their implementation in the near future (i.e., more frequent inclusion of perinatal exposure in chronic studies, better assessment of endocrine-related responses in subchronic studies). As discussed above, the recommendation to use alternative models that might be more sensitive for a particular effect is already considered in the designing of NTP studies. Other decisions, such as which rat strain will replace the F344/N, have not been made yet, but the issues raised at this workshop will play a significant role in selection of a new strain. The NTP appreciates the active participation and open debate by workshop attendees in helping identify solutions to issues facing the program. During 2007, NTP scientists will meet to consider the feasibility and logistical, scientific and practical considerations of the various workshops’ recommendations, and address modifications to the testing program. Announcements of significant program changes will be made in future workshop reports, the NTP newsletter, and at meetings of its various advisory committees. The NTP is confident that these changes will strengthen its research and testing activities and enhance the scientific information available for making sound decisions that protect public health.

## References

[b1-ehp0115-001351] Ahlers I, Solar P, Buresova A, Ahlersova E (1998). Very low sensitivity of Wistar:Han female rats to chemocarcinogens in mammary carcinogenesis induction. Neoplasma.

[b2-ehp0115-001351] Barrett JR (2006). NTP multigenerational study of environmental estrogens. Environ Health Perspect.

[b3-ehp0115-001351] Bordelon NR, Chhabra R, Bucher JR (2005). A review of evidence from short-term studies leading to the prediction that diazo-aminobenzene (1,3-diphenyltriazine) is a carcinogen. J Appl Toxicol.

[b4-ehp0115-001351] Chhabra RS, Bucher JR, Haseman JK, Elwell MR, Kurtz PJ, Carlton BD (1993a). Comparative carcinogenicity of 5,5-diphenylhydantoin with or without perinatal exposure in rats and mice. Fundam Appl Toxicol.

[b5-ehp0115-001351] Chhabra RS, Bucher JR, Haseman JK, Elwell MR, Kurtz PJ, Carlton BD (1993b). Comparative carcinogenicity of poly-brominated biphenyls with or without perinatal exposure in rats and mice. Fundam Appl Toxicol.

[b6-ehp0115-001351] Cook JC, Klinefelter GR, Hardisty JF, Sharpe RM, Foster PM (1999). Rodent Leydig cell tumorigenesis: a review of the physiology, pathology, mechanisms, and relevance to humans. Crit Rev Toxicol.

[b7-ehp0115-001351] Gold LS, Manley NB, Slone TH, Ward JM (2001). Compendium of chemical carcinogens by target organ: results of chronic bioassays in rats, mice, hamsters, dogs, and monkeys. Toxicol Pathol.

[b8-ehp0115-001351] Holm M, Rajpert-De Meyts E, Andersson AM, Skakkebaek NE (2003). Leydig cell micronodules are a common finding in testicular biopsies from men with impaired spermatogenesis and are associated with decreased testosterone/LH ratio. J Pathol.

[b9-ehp0115-001351] Irwin RD (2006). A review of evidence leading to the prediction that 1,4-butanediol is not a carcinogen. J Appl Toxicol.

[b10-ehp0115-001351] King-Herbert A, Thayer K (2006). NTP Workshop: Animal Models for the NTP Rodent Cancer Bioassay: Stocks and Strains—Should We Switch?. Toxicol Pathol.

[b11-ehp0115-001351] NTP (2005a). NTP Workshop: Animal Models for the NTP Rodent Cancer Bioassay: Strains & Stocks—Should We Switch?. http://ntp.niehs.nih.gov/go/17355.

[b12-ehp0115-001351] NTP (2005b). Report on Carcinogens. http://ntp.niehs.nih.gov/go/19914.

[b13-ehp0115-001351] NTP (2005c). Roadmap to Achieve the NTP Vision (10 March 2005). http://ntp.niehs.nih.gov/go/vision.

[b14-ehp0115-001351] NTP (2006a). Draft NTP Technical Report on the Toxicology and Carcinogenesis Study of Genistein (CAS No. 446–72–0) in Sprague-Dawley Rats (Feed Study). http://ntp.niehs.nih.gov/go/28023.

[b15-ehp0115-001351] NTP (National Toxicology Program) (2006b). NTP Technical Report on the Toxicology and Carcinogenesis Studies of 2,2’,4,4’,5,5’-Hexachlorobiphenyl (PCB 153) (CAS No. 35065–27–1) in Female Harlan Sprague-Dawley Rats (Gavage Studies). Technical Report 529.

[b16-ehp0115-001351] NTP (National Toxicology Program) (2006c). NTP Technical Report on the Toxicology and Carcinogenesis Studies of 2,3,7,8-Tetrachlorodibenzo-*p*-Dioxin (TCDD) (CAS No. 1746–01–6) in Female Harlan Sprague-Dawley Rats (Gavage Studies). Technical Report 521.

[b17-ehp0115-001351] NTP (2006d). NTP Workshop: Biomarkers for Toxicology Studies, 20–21 September 2006, Raleigh, NC. http://ntp.niehs.nih.gov/go/20940.

[b18-ehp0115-001351] NTP (2006e). NTP Workshop: Hormonally Induced Reproductive Tumors—Relevance of Rodent Bioassays, 22–24 May 2006, Research Triangle Park, NC. http://ntp.niehs.nih.gov/go/18592.

[b19-ehp0115-001351] NTP (2006f). Testing Status of 2,2’4,4’-Tetrabromodiphenyl Ether (DE-47). http://ntp.niehs.nih.gov/see“Testing.

[b20-ehp0115-001351] Russo IH, Russo J (1998). Role of hormones in mammary cancer initiation and progression. J Mammary Gland Biol Neoplasia.

[b21-ehp0115-001351] Simanainen U, Adamsson A, Tuomisto JT, Miettinen HM, Toppari J, Tuomisto J (2004). Adult 2,3,7,8-tetrachlorodibenzo-*p*-dioxin (TCDD) exposure and effects on male reproductive organs in three differentially TCDD-susceptible rat lines. Toxicol Sci.

[b22-ehp0115-001351] Suttie AW, Dinse GE, Nyska A, Moser GJ, Goldsworthy TL, Maronpot RR (2005). An investigation of the effects of late-onset dietary restriction on prostate cancer development in the TRAMP mouse. Toxicol Pathol.

[b23-ehp0115-001351] Tam NN, Nyska A, Maronpot RR, Kissling G, Lomnitski L, Suttie A (2006). Differential attenuation of oxidative/nitrosative injuries in early prostatic neoplastic lesions in TRAMP mice by dietary antioxidants. Prostate.

[b24-ehp0115-001351] U.S. EPA (1998). Health Effects Test Guidelines. OPPTS 870.3800. Reproduction and Fertility Effects. http://www.epa.gov/opptsfrs/publications/OPPTS_Harmonized/870_Health_Effects_Test_Guidelines/Series/870-3800.pdf.

[b25-ehp0115-001351] U.S. EPA (2000). Atrazine: Evaluation of Carcinogenic Potential (memorandum). http://www.epa.gov/opptsfrs/publications/OPPTS_Harmonized/870_Health_Effects_Test_Guidelines/Series/870-3800.pdf.

[b26-ehp0115-001351] U.S. EPA (2006). Information on the Endocrine Disruptor Screening Program. http://www.epa.gov/oppsrrd1/reregistration/REDs/atrazine_ired.pdf.

